# Exploring Health Information Sharing Behavior of Chinese Elderly Adults on WeChat

**DOI:** 10.3390/healthcare8030207

**Published:** 2020-07-10

**Authors:** Wei Wang, Xin Zhuang, Peng Shao

**Affiliations:** 1 School of Media and Law, NingboTech University, Ningbo 315100, China; donnaww81@nit.zju.edu.cn; 2 National Science Library of Chinese Academy of Sciences, Chinese Academy of Sciences, Beijing 100190, China; zhuangxin@mail.las.ac.cn; 3 School of Humanities, Zhejiang University of Technology, Hangzhou 310014, China

**Keywords:** social media, health information behavior, elderly adults, health communication

## Abstract

WeChat has increasingly become an important platform for users to acquire and share health information in China. However, little is known about elderly adults’ sharing behavior. This study aims to explore the characteristics and influencing factors of health information sharing behavior among Chinese older adults on WeChat, with the method both of questionnaire survey (*N* = 336) and in-depth interviews (*N* = 40). The study finds that sharing health information, mainly represented by four specific methods of forwarding, consulting, replying, and posting, has become an important part of the daily life of elderly adults on WeChat. Social media provide a good opportunity for the flow of health information. However, the purpose to share health information of Chinese older adults is mainly based on relationship maintenance more than real information support; they share health information to friends and relatives first, then to spouses and children, which does not follow the trust model as usual. Experience in online health information, authority orientation, and relationship orientation is positively associated with health information sharing behavior, however, there is no significant correlation between perceived health information credibility and health information sharing behavior. Moreover, social and cultural factors are the important explanation mechanism.

## 1. Introduction

The immense popularity of social media use among people from varying demographic groups has attracted the attention of communication scholars [1,2]. China has entered into an aging society, and under the “Healthy China” national strategic system, it has become an important and urgent issue to promote health communication among Chinese elderly adults. In the social media platform, social interaction facilitates the sharing of health information and opens up new paths for health communication. At present, social media is gradually infiltrating into the elderly group, and under the cultural background of “Yang Sheng (Keeping in good health).” In China, more and more older adults regard health information seeking and sharing behavior as their daily life on social media, especially on WeChat. It thus becomes important to study factors associated with older adults’ health information sharing behavior.

Despite the proliferation of research on health information seeking behavior (HISB), health information sharing behavior remains under-researched, especially for Chinese elderly adults in the social media context. Very little research with local perspectives has been conducted on the influencing factors of health information sharing behavior. Chinese elderly adults are deeply influenced by traditional culture, and are also in the information age environment, under the background of the turbulence of traditional and modern, so does the health information sharing behavior of the elderly adults have subcultural characteristics, which are different from other groups? It is important to fill this research gap since it expands the scope of the prior literature and theory.

Bandura’s social learning theory attempts to predict and explain behavior with several key concepts, one of them is self-efficacy which influences all aspects of behavior [3]. The concept of self-efficacy was introduced into general health practice areas such as smoking, weight control, alcohol abuse, and exercise, but health information sharing behaviors were not included. The study of credibility has a long history in the academy [4]. Aristotle listed good sense, good moral character, and goodwill as source qualities [5]. Much of Hofland’s research focused on the credibility of communicators [6], credibility has been examined as a factor influencing message receivers’ perceptions, attitudes, and behaviors. At the same time, human behavior is social and cultural. Cultural group characteristics may be associated with health-related beliefs, attitudes, and behaviors [7]. Under collectivism culture and individualism culture, people often have different behaviors, for example, people will have more sharing behavior and reciprocity activity just for “Mianzi” (face) in collectivism culture [8].

Therefore, this study will integrate social cognition theory, media credibility theory, and some theoretical resources of local psychology to explore the characteristics and influencing factors of health information sharing behavior among Chinese older adults on WeChat. In the sections that follow, we define health information sharing behavior as the purposive transmission of health information to others in the context of social media. Next, we review research on the self-efficacy, media credibility, social orientation, and the potential relationship with health information sharing behaviors, we then explain the influencing mechanism. This study shows a holistic picture of the characteristics and influencing factors of health information sharing behavior of Chinese elderly adults. It offers a theoretical contribution to the literature of health information sharing behavior by incorporating the social and cultural angle, and its findings are especially relevant for the development of the health information industry, the government and public health departments, and IT developers and designers.

## 2. Background Literature

### 2.1. Social Media and Information Sharing

Social media offers an excellent medium for communication, connecting, academic uses, psychosocial well-being, and so on [9,10]. Social media have not only enabled users to share numerous forms of content [11,12,13], but also provided them with new ways to organize, manage, and discover that content [14].

In the health communication field, social media are channels for sharing health information, information flow and relationship interaction promote each other on social media [15]. Information through relationship spreads faster and more effective, which brings convenience to health information exchange and opens up a new path for health communication. A recent survey suggested that, of the 8% of Internet users in the USA who indicated that they had post health-related information online, nearly half of them said they were sharing their personal health experience [16]. Medlock and colleagues found that seniors also take the Internet as a preferred and most popular source of health information, despite concerns over the quality of online information [17]. Scholars believed that social media have great potential to support information searching and decision making on self-care and health-related issues [18]. Nonetheless, the proliferation of new media in health care also poses several problems and challenges, especially, the quality of the health-related information on social media is far from perfect, which is often inconsistent, misleading, and not trustworthy [19].

### 2.2. Health Information Sharing Behavior

Information sharing is an important field of information behavior research, which refers to voluntary behaviors that make information available to others. Wang believes that with the development of information technology, information sharing has become the main way for users to obtain and use information [20]. The health information sharing behavior in this article refers to the information interaction process of transferring health information from one party to other parties through online communication and interaction. In this study, we aim to explore the characteristics and influencing factors of health information sharing behavior of the Chinese elderly adults. The following research questions are proposed:

Q1: What are the specific behaviors of the elderly in China to share health information?
Q2: To whom do Chinese elderly adults share their health information?

### 2.3. Self-Efficacy and Health Information Sharing Behavior

Social learning theory believes that self-efficacy is closely related to the maintenance or change of individual behavior [21]. The concept of self-efficacy relates to beliefs about capabilities of performing specific behaviors in particular situations. Self-efficacy will vary greatly depending on the particular task and context which confront him or her [22]. For all health-related areas studied, self-efficacy appears to be a consistent predictor of short- and long-term success, which means strong associations between self-efficacy and progress in health behavior change and maintenance [23]. The concept of self-efficacy has also been applied to knowledge management to validate the effect of personal efficacy belief in knowledge sharing, that is knowledge sharing self-efficacy (KSSE). Several researchers have employed KSSE to examine its effect on knowledge sharing intention. For instance, Bock and Kim propose that self-efficacy could be treated as a major factor of the self-motivational source for knowledge sharing [24].

Guo and his colleagues believed that sharing behavior is related to self-efficacy [25]. It has also been found that greater self-efficacy led to higher levels of online health information sharing [26]. The performance achievements obtained by individuals through positive experience are the most powerful source of their sense of self-efficacy, that is, users with more experience in online health information are more likely to have higher health information self-efficacy, and are more likely to have health information sharing behavior. However, Crook and his colleagues developed a theoretical model of health information sharing behavior. It was believed that individuals with more Internet usage experience would have high health literacy, and individuals with high health literacy often have more negative evaluations of health information and would do less health information sharing behavior [27]. Internet usage experience may also lead to information overload problems, leading to its negative attitude to health information, which will negatively affect health information sharing behavior. Through the above analysis, the relationship between online health information experience and health information sharing behavior is different, and there is no empirical data support for elderly adults. However, according to the actual situation in China, the Internet usage level and experience of the elderly in China were generally low [28], they may not have suffered an information overload problem. Therefore, the research hypothesis is put forward:

**Hypothesis 1**  **(H1).** *Chinese elderly adults with more experience in online health information are more likely to share health information on WeChat*.

### 2.4. Media Credibility and Health Information Sharing Behavior

Media credibility has traditionally been an important topic in communication research. It refers to the degree of trust expressed by the audience after subjective judgment and evaluation in the long-term media usage, and is the result of the audience’s assessment of the quality of the media [29]. For example, users’ evaluation of online health information relies heavily on peripheral cues and contextual factors [30]. Media credibility is regarded as a multi-level structure, which can be subdivided into three categories: source credibility, information credibility, and channel credibility [31]. Scholars examined various types of credibility relevant to website, including the credibility of the website as a whole, the credibility of the website sponsor, and message credibility [32]. Zhou and Yan argued that there were so many studies focused on peripheral factors such as information source, media, and audience characteristics in the research on media credibility, but the core element of information credibility is under-researched [33]. Zhang also believed that there were significant differences in credibility between different media and different types of information [34]. The credibility of the content is mainly composed of information source and the gatekeepers in the traditional media, but the source of health information on social media is diversity and hard to trace, therefore, the study on social media credibility, can be transferred from the source and media credibility to the study of information credibility, therefore, the study reported here focused on message (health information) credibility.

Evaluating the quality of health information has been a major challenge for online health consumers [35,36,37], which may affect their subsequent behavior. Many studies have been conducted and confirmed that the credibility perception of online health information will affect the subsequent behaviors of users, such as the discussion of health problems and the sharing of health information [38,39,40]. Using the data from the 2007 Health Information National Trends Survey, Hou found that trust in online health information was a significant predictor of conducting health-related activities via the Internet [41]. Huh, Delorme, and Reid found that the higher level of trust in online drug-related information, the more likely one would engage in three types of behaviors, including communicating with doctors, talking with others, and seeking more health-related information [42]. Additionally, Chen and Sharma drew upon the social capital theory and concluded that the level of trust in other users on social networking sites was positively associated with the degree of self-disclosure [43]. Chung and his colleagues found that useful and reliable information will promote the information sharing behavior of social network users [44].

However, other scholars have come to different conclusions. For example, Kim suggested that the impact of information quantity on trust and willingness to share information has not been researched thoroughly [45]. Chang and Dong believe that the objectivity, timeliness, and interest of information will positively affect information sharing behaviors through users’ information sharing intentions, while the relevance and reliability of information have no significant influence on the information sharing intention [46]. Li also believed that the elderly WeChat users in China had relatively little demand for information reliability, and the elderly mainly used WeChat as an auxiliary way to communicate with their friends, and they could not obtain more useful information from WeChat [47]. It can be seen that there are different opinions about the relationship between health information credibility and health information sharing behavior. Combined with the findings of previous interviews in this study, the authors believe that the situation of the elderly in China may be more consistent with the findings of the Chinese scholars.

**Hypothesis 2** **(H2).** *Credibility of health information may not significantly affect the health information sharing behavior of elderly WeChat users.*

### 2.5. Social Orientation and Health Information Sharing Behavior

Information behavior has social and cultural characteristics, which means that the specific information behavior is affected by the social ideology and the social environment. The characteristics of the collectivist and individualistic cultures in Asia and North America are much different. Asian culture features the values of family-prioritized, obedience, duty, and in-group harmony [48]. The elderly people were deeply affected by traditional Chinese culture, and therefore have a behavioral tendency that is appropriate to cultural values. Local psychologists argue that, when studying Chinese psychology and behavior, the influence of “social orientation” could not ignored. Social orientation values will have an impact on social interaction and behavior. Specifically, the orientation of Chinese society mainly includes four kinds of orientations: First, the family orientation is essentially a kind of family collectivism, which includes eight behavioral tendencies such as focusing on the family, striving for the family, and distinguishing between inside and outside. Secondly, the relationship orientation is mainly expressed in the formalization of the relationship (role playing), relationship interdependence (reciprocity) and relationship harmony, in order to pursue harmony, the harmony strategy is often adopted to save others’ face and avoid possible conflicts. Thirdly, the authority orientation, is mainly manifested in the individual’s sensitivity to and worship of authority, and the natural tendency to trust in authority. Fourth, the orientation of others refers to the psychological and behavioral susceptibility to others, trying to be consistent with others, hoping to leave a good impression with others [49]. It has been found that the most common motive for sharing health information was the desire to help others [26]. During the interview, it was found that social orientation has a certain relationship with the health information sharing behavior of the elderly people. However, respondents mentioned orientation of others less frequently. The authors believe that the essence of orientation of others belongs to relationship orientation, which is an inevitable extension of relationship orientation. Based on this, this study divides the social orientation into three dimensions: relationship orientation, family orientation, and authority orientation. The research hypotheses are put forward:

**Hypothesis 3-1** **(H3-1).** *Relationship orientation should significantly affect the health information sharing behavior of elderly adults on WeChat.*

**Hypothesis 3-2** **(H3-2).** *Authority orientation should significantly affect the health information sharing behavior of elderly adults on WeChat.*

**Hypothesis 3-3** **(H3-3).** *Family orientation should significantly affect the health information sharing behavior of elderly adults on WeChat.*

## 3. Materials and Methods

This study used a combination of qualitative and quantitative research methods. At the beginning, we explored unknown variables through the in-depth interview method. Then, the questionnaire survey method was mainly used to collect data and test hypotheses and verify theories. At last, the experience materials in the in-depth interviews were used again to deeply explain the influencing factors and influencing processes of the health information sharing behavior of elderly users on WeChat.

In terms to the definition of “elderly adults”, according to China’s retirement policy, that is, men over 60 years old and women over 55 years old [50]. According to the existing research literature, senior citizens living in cities with higher income and education levels are more likely to seek health information on the Internet [51], and social media users among elderly people mainly live in the city and have a higher level of education [52], therefore, this study mainly selects senior college students as the specific research participants.

As for research ethics, we considered two aspects. First, we informed the two colleges the aim of the research, the research process, and ethical issues. Secondly, the participants themselves were informed of the principles of voluntary participation, anonymity, anticipated benefits, and different ethical considerations concerning participation. Specifically, all participations in the survey were kept voluntary and anonymous, no personal identifiable information except age, gender, and some other insensitive demographic information were obtained from the study. The in-depth interviews were recorded with prior consent, and the materials were used anonymously. Article 40 in the Constitution of the People’s Republic of China stipulates that the freedom and privacy of correspondence of citizens in China shall be protected by law, which makes clear the protection of the freedom and privacy of communication [53]. The study did not breach any research integrity guidelines in China.

In semi-structured interviews, participants for this study were recruited from senior college students by snowballing, namely through peer recommendation to successively organize the sample. Forty interviews were conducted and the distribution of participants by gender was balanced—22 women and 18 men. On average, interviews lasted for 1 h. Then, the interview data were analyzed through content analysis, which assisted in the demonstration of some research findings and conclusions in quantitative research.

At the time of the formal questionnaire survey, two colleges were selected in Zhejiang province in southeast China. The survey is a written survey, the questionnaire was conducted in a face-to-face written manner, teachers assisted the researchers to distribute them in the classroom and recall it on the spot. At last 500 questionnaires were distributed through systematic sampling according to student number and 336 valid questionnaires were recovered. The effective recovery rate was 67.2%. The questionnaire had good reliability and validity.

There are 4 items for measuring health information sharing behaviors, which are coded 1–4 from “frequent” to “never” according to the behavior level. Responses were summed to create the health information sharing behavior index (*а* = 0.78, M = 3.07, SD = 0.54).

Relationship orientation was measured according to Yang’s illustration [49] by 4 items (e.g., “if someone sent information to me, I will send another information to him or her later”; *а* = 0.74). Participants answered on a Likert-type format ranging from 1 (strongly agree) to 4 (strongly disagree). Authority orientation was measured by 3 items (e.g., “I trust the government and public institutions”; *а* = 0.78). Participants answered on a Likert-type format ranging from 1 (strongly agree) to 4 (strongly disagree). Family orientation was measured by 3 items (e.g., “the happiness of children and grandchildren is the biggest happiness for me”; *а* = 0.75). According to factor analysis, the KMO (Kaiser-Meyer-Olkin) value is 0.815, the chi-square value of Bartlett sphericity test is 967.811, and the statistical test is significant at the level of 0.000. So, the scale items are suitable for factor analysis. The factor analysis results showed that 10 variables were condensed into 3 factors, and the cumulative variance contribution was 69%. The common factor variance of each item was approximately above 0.6.

Online health information experience was measured by 3 items, “browsing professional websites, portal sites, Baidu-search respectively to obtain health information”. Participants answered on a Likert-type format ranging from 1 (often) to 4 (never). Responses were summed to create the network health information experience index (*а* = 0.77, M = 2.86, SD = 0.68)

Health information credibility was measured by 3 items following previous research on this topic [54], participants were asked to rate the degree to which the health information they viewed was poor accuracy, inconsistent, insufficient reliability, respectively. Participants answered on a Likert-type format ranging from 1 (strongly agree) to 4 (strongly disagree). Responses were summed to create the health information credibility index (*а* = 0.79, M = 1.98, SD = 0.43)

## 4. Results

Demographic variables, regression analysis was employed to examine the proposed hypotheses and research questions. Data were analyzed using SPSS 10.01 (SPSS Inc., Chicago, IL, USA). Descriptive statistics for the participants and the main variables are showed in Table 1.

The study found that the types of health information sharing behaviors elderly users on WeChat mainly include are forwarding, inquiring, posting, and replying information. Among them, forwarding information is the most common, posting and replying information is relatively rare. For the entire sample, 16.7% (*n* = 336) of elder adults had “forward” health messages more often on WeChat. Followed by “reply” information (4.5%, *n* = 336), “post” information (3%, *n* = 336), “inquire” information was only mentioned in 2.1% (*n* = 336). For the complete sample, the mean number of “forward” was 29.85 (SD = 213.099), and the mean number of “reply”, “post”, “inquire” information was 20.12 (SD = 109.349), 20.12(SD = 109.349), and 20.12(SD = 109.349), respectively (See Table 2).

Older adults always lack confidence in their ability to evaluate online health information credibility [36]. Sometimes they forward some health information to their friends, but it was only for them to make a reference and it could not be fully believed. Just like respondent Li said, “I had forwarded health information to my friends, but not making too many recommendations, they can make the decision by themselves”.

Forwarding behavior may related to the factor of the different stage, respondents. Huang said when he started to learn how to use WeChat, he was much likely to forward information (including health information). After forwarding the information, other people’s praise behavior made him think that he was still closely following the trend of the times (on the edge of popular), but over time, as the freshness reduced, the behavior of forwarding health information sharing was less. The findings of this study echo the previous research that time lapse is one of the reasons why older males untag photos on Facebook [55]. But in this study, it is mainly due to the professional consideration of health information. Due to the professionalism of health issues, elderly people are often more cautious about inquiring and replying to health information, and inquiring health information is more than posting health information.

**Figure 1:**
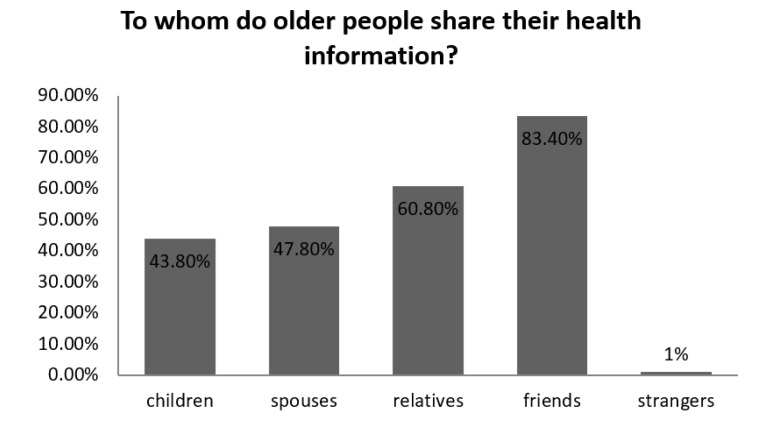
To whom older people share their health information.

In addition, the health information sharing behavior is a selective interpersonal interaction process, including the choice of sharing objects and sharing content. According to the statistical analysis of this study, in terms of health information sharing objects, the elderly people mainly shared health information with friends and relatives, followed by spouses and children (see Figure 1).

For example, one participant named Shao explained that “children have a higher level of education and have higher ability to understand and judge health information than me.” Some respondents said that they would occasionally forward health information to their children, only when reminding them to drink more water, enjoy more sleep, exercise, and other health information, but this was mainly for emotional connection needs, rather than the actual information effect.

According to the linear regression (see Table 3), the F statistic of the overall test of the regression model reached the significant level (F = 13.865.791, *p* < 0.001), and the explanatory ability of the independent variable to the health information sharing behavior was 25.3%.

Online health information experience (=0.417, *p* < 0.001) has significant influence on health information sharing behavior, but health information credibility perception has no significant influence on health information sharing behavior. In terms of social orientation, authority orientation (=0.119, *p* < 0.05) and relationship orientation (=0.109, *p* < 0.05) have significant influence on health information sharing behavior, but family orientation has no significant influence on health information sharing behavior. In addition, women share more health information than men.

## 5. Discussion

The data showed that the social media platform provides a good opportunity for the flow of health information. Health information sharing has become an important part of the daily communication among elderly people on WeChat. These health information sharing activities are mainly represented by four specific methods of forwarding, consulting, replying, and posting. However, elderly people are more likely to participate in health information interaction by forwarding health information, that means, they often use the intermediary of forwarding identity rather than content contributor identity in the information sharing behavior on the WeChat platform, they prefer to observe the behavior of others, while the content of their own is less updated (answering Q1). Our findings revealed that elderly adults forward health information mainly based on the direct forwarding of the original text, which benefits from the convenience of one-click forwarding function on WeChat. Forwarding behavior may relate to the factor of the different stage, as the freshness is reduced, the behavior of forwarding health information will be less than the new learners. Due to the professional consideration of health information, older people tend to be more cautious in asking for help or replying health information.

In the field of knowledge sharing research, interpersonal trust relationships promote knowledge (information) sharing behavior, which means that as trust relationships increase, people generally have more possibilities of information sharing behavior. Wang and Liu divided into four kinds of trust in daily life: social trust, acquaintance trust, friend trust, and family trust [56]. Among these four types of trust, family members have the strongest trust, followed by friends, acquaintances, and social trust as the weakest trust. However, this study found that health information sharing behavior of the elderly people on WeChat, which does not simply spread with the above diffusion of the trust model. On the one hand, for the sake of safety, elderly adults are less likely to add strangers as friends and interact with them, and accordingly, there is less health information sharing behaviors, But as far as acquaintances are concerned, the elderly tend to share health information with peers such as friends and relatives rather than their spouses and children (answering Q2). Older adults do not share health information with family members first, but with their friends. The reason for this can be explained by the culture and digit feedback theory which means children always have more ICT (Informational Communications Technology) skills and health information literacy than their parents, which in turn can help parents improve these skills.

Integrating social learning theory, media credibility theory, and the concept of social orientation of native psychology, the study focuses on the influencing factors of health information sharing behavior, and more specifically, to explore how personal experience and self-efficacy, perception of information credibility, and the three types of social orientation, were related to health information sharing behavior. This study revealed that online health information experience (H1), relationship orientation (H3-1), and authority orientation (H3-2) were positively associated with health information sharing behavior. However, perception of health information credibility (H2) and family orientation (H3-3) were not found to be related to health information sharing behavior (see Table 4).

Firstly, this study confirmed that users with rich experience in online health information are more likely to share health information (H1), which may be related to the mechanism of self-efficacy. In this study, online health information experience can be regarded as the positive experience of the elderly adults to increase their self-efficacy. Through the accumulation of these positive experiences, the self-efficiency of the elderly people can be improved, which in turn affects their health information behavior. At the same time, users with rich experience in online health information should benefit from the spiritual rewards after sharing behavior. The elderly people always regard information sharing as a kind of “gift”, from which they can experience “successful”, “helpful”, and other feelings, thus generating a sense of pleasure and satisfaction. Just like participant Gao said “I would be happy to see ‘thumb up’ (likes from receivers) after forwarding health information to my friends, which make me believe I can provide more useful health information”. This kind of good social feedback from others would improve the sense of health information self-efficacy.

We did not find any significant relationship between the credibility of health information (H2) and user intentions health information sharing behavior of the elderly adults. Elderly people are less sensitive about the usefulness and reliability of health information, they would rather adopt heuristic strategies when sharing health information, than by cognitive strategies such as judging the quality of health information itself.

Bilchik and Heyman argued credibility ratings vary substantially among different cultures and within different social systems [57]. It is essential to conduct media credibility studies in Eastern countries like China, which has a fairly unique, say, authority-directed collectivism culture. This is also why we introduction the social orientation variable in this study to explain the locality of health information sharing behaviors of the elderly people in China. The study results confirmed the impact of the social orientation of the Chinese elderly people on their health information sharing behavior. Specifically, relationship orientation and authority orientation can significantly affect their health information sharing behavior, but we did not find any significant relationship between family orientation and their health information sharing behavior.

The study results suggest that health information sharing behavior is directly influenced by relationship orientation (H3-1). The findings of this study echo the previous research that the intensity of the relationship had a significant impact on the willingness to share health information on the microblog [58]. Frequent interaction and intimate relationship between users led to a stronger willingness to share information. The findings are also consistent with several recent studies that have emphasized the emotional and social values exert partial influence in predicting users’ behavior intention [59,60,61]. Social interaction influences a user’s flow experience, which is the intrinsic aspect of his behavior (active participation) [62,63]. The basic and priority status of “relationship network”, but not the “information flow” is fully reflected in the health information sharing behavior of elderly adults. The health information sharing on the WeChat platform promotes the exchange of emotion and relationship. As Zhai once revealed that the same kind of exchange enhances emotions, the higher the degree of homogeneity of exchange, the stronger the relationship builds [64]. The health information sharing behavior in social media is not only the process of information acquisition and utilization, but also the process of interaction and socialization of others. Although the health information sharing behavior of the elderly adults is an independent individual behavior, it is also embedded in the relationship interaction, and affected by cultural environment and social relations.

As the respondent Tong said, “If a friend once forwarded me a health message, as a feedback, usually I will forward a health message to her afterwards,” and “if the health message forwarded by a friend is of poor quality, I would not directly point it out.” It can be seen that the relationship orientation of the elderly adults is represented in the “gift-like” health information sharing behavior of “you send one message to me, and I will forward another to you” on the WeChat platform, and there is the phenomenon of turning a blind eye to the problem of health information quality and not saying anything. That is why Guess et al. found that people who were over 65 had the highest rate of fake news sharing than other age groups [65]. The study of information behavior of the elderly should not ignore the unique psychological factors of the elderly. According to socioemotional selectivity theory (SST), elderly adults are more aware of the limitation of time, tend to dissolve negative emotional experience in life, and pay more attention to the positive side of the emotion regulation goal than knowledge acquisition goal [66].

In addition, authority orientation could significantly affect the health information sharing behavior of elderly adults (H3-2), that is, the older people who believe in the authoritative opinions are more likely to share health information on WeChat. This can be explained by the transfer of the trust mechanism. We can see that trust in authority will shift from offline to online, elderly people with authoritative orientation are more likely to trust medical institutions or the media institutions, then they will more likely to pay attention to the health information released by the professional medical institution on WeChat, accordingly will more likely to share health information to others, to reflect a kind of responsible reciprocity motivation. This is consistent with the research conclusion of knowledge sharing behavior, in which scholars have introduced the theory of social impact into the study of knowledge sharing behavior, thinking that people accept social influence because individuals want to adapt to the influence of the organization. Viewpoints, social recognition, and group norms have a significant impact on users’ knowledge sharing behavior [67].

As for family orientation, it could not significantly affect their health information sharing behavior (H3-3). Although the elderly has more family orientation, this “home culture” does not mean that they will have more health information sharing behaviors. As mentioned above, they often share less health information to their children due to the mechanism of cultural feedback or digital feedback.

## 6. Conclusions

Health information sharing behavior of older adults on social media has not received much attention from health communication scholars. This study, designed to fill that gap, examined the traits of Chinese older adults’ health information sharing behavior and the factors which were related to this behavior, using a sample of 336 elderly adults aged 60 to 75, and also with 40 elderly adults in the in-depth respondents. This proposed study has contributed significantly to both theory and practice.

### 6.1. Theoretical and Practical Implications

Health information sharing behavior of older adults on social media has not received much attention from health communication scholars. This study, designed to fill that gap, examined the traits of Chinese older adults’ health information sharing behavior and the factors which were related to this behavior, using a sample of 336 elderly adults aged 60 to 75, and also with 40 elderly adults in the in-depth respondents. This proposed study has contributed significantly to both theory and practice. The findings contribute to extended prior literature in the field of health communication, information behavior, gerontology, and new media in different ways. This study underscores the importance of social orientation as determinants of health information sharing behavior of Chinese elderly adults.

The study offers a theoretical contribution to the literature of health information sharing behavior by incorporating the social and cultural angle. In the past, health information behavior research was based on rational behavior theory and using Western scales for microscopic research [68]. This study highlights the important role of social and culture, and introduces the local macro factors of “social culture” to explore its influence on the health information sharing behavior of the elderly people. At the same time, the “information” and “relationship” variables were integrated to analyze their impact on health information sharing behavior on social media, Overall, this study extends the scope of health information sharing behavior research and paves the way for future research in this area.

It yields important insights for the development of the health information industry, the government and public health departments, and IT developers and designers. Specifically, in view of the current health information sharing behavior of the elderly and the conclusions of this study, it is necessary to purify the health information ecological environment in social media. Public health service organizations must not only make full use of emerging technology platforms such as social media to bring themselves to the foreground, but also seize the opportunities of health information dissemination, reverse the gloomy situation of “bad money drives out good money”. Medical institutions and media agencies should also participate in the health information dissemination campaign. They should improve media literacy and health literacy respectively [69,70]. Government departments and professional agencies should act as information disseminators and authoritative news media. It should become the main channel for information dissemination to improve the accuracy, completeness, and continuity of online public health information as soon as possible.

It also should pay attention to the relationship orientation and actively play the role of opinion leaders of the same age group of the elderly. Opinion leaders in these peer groups can frequently influence the attitudes and behaviors of others in terms of the adoption and use of new media, the perception of credibility of health information, and specific health information behaviors. Therefore, health communication organizations should use social media to identify and communicate medical professional opinion leaders, public opinion leaders, and grassroots opinion leaders to start a new era of health communication.

From a micro level, it is important to cultivate and improve the electronic health information literacy of the elderly individuals. Helping the elderly to improve the abilities of individuals to search, discover, understand, and evaluate the health information on electronic resources, and use health information to solve their health problems and improve their health level.

### 6.2. Limitations and Future Research

The present study has significantly contributed to literature and theory. However, the study also has a few limitations. One limitation is that the respondents are all senior college students, their education level is higher than the overall level of the general elderly. Although previous studies have found that they are more likely to be exposed to social media and seek online health information, the results cannot be generalized. 

Secondly, the study tries to introduce “social culture” factors to explore the influence of the behavior of Chinese elderly adults, but in the specific operation process, it was only manipulated into three variables (family orientation, relationship orientation, and authoritative orientation), so the result can only see the tip of the iceberg of social culture.

Thirdly, there are various types of health information in social media, including health care information, specific disease information, nutrition information, and rumor information. For different types of health information, people are likely to adopt different information sharing behaviors, but in this study, it did not distinguish between these health information and information sharing behaviors.

There are some recommendations for future research. First, with the further penetration of social media among elderly people, the scope of respondents should be expanded in the future, and random sampling should be conducted by household survey as far as possible to improve the generalizability of the findings. Secondly, the influencing mechanism of social and cultural factors to health information sharing behavior is still inadequate, in the future, we can do a more in-depth interview method to complement this study. At last, future studies may focus on a certain type of health information or a comparative study of other types of health information to explore the health information sharing behavior, which may find other factors that could potentially influence the behavior.

## Figures and Tables

**Table 1:** Descriptive statistics of variables analyzed (*N* = 336).

Variables	Range	M(SD)	%
Age in years	60–75		
Female (vs. male)			62.8%
Health status (with chronic disease)			65.7%
Education	1–5 (from illiteracy to junior college or above)	5.39 (0.77)	
Personal monthly income	1–6 (from￥500–1000 to￥6500 yuan or more)	4.9 (1.08)	
online health information experience	1–4 (from often to never)	2.86 (0.68)	
Health information credibility	1–4 (from bad to good)	1.98 (0.43)	
Relationship orientation	1–4 (from Very much in line to not at all)	2.10 (0.48)	
Authority orientation	1–4 (from Very much in line to not at all)	1.87 (0.49)	
Family orientation	1–4 (from Very much in line to not at all)	1.61 (0.47)	
Health information sharing	1–4 (from often to never)	3.07 (0.54)	

**Table 2:** Descriptive statistics of the concrete health information sharing behaviors.

Health Information Sharing	More Often (%)	Sometime (%)	Occasionally (%)	Never (%)	M (SD)
Forward health information	16.7	50.3	19.9	13.1	2.36 (0.89)
inquire health information	2.1	38.7	31.8	27.4	3.14 (0.69)
reply health information	4.5	34.7	29.5	31.3	3.15 (0.77)
Post health information	3	33.6	32.1	31.3	3.24 (0.70)

**Table 3:** Standardized coefficients from linear regression of health information sharing behavior (*N* = 336).

Variable	SE (Standard Error)	*β* (Beta)	*t* (t Test Value)	Sig. (Significance)
constant	0.209		7.207	0.000 ***
Gender (female)	0.057	0.102	1.999	0.046 *
Health status (with chronic disease)	0.051	0.024	0.497	0.620
Personal monthly income	0.026	0.018	0.349	0.727
Health information credibility	0.026	−0.009	−0.190	0.849
online health information experience	0.040	0.417	8.354	0.000 ***
Authority orientation	0.065	0.119	2.037	0.042 *
Family orientation	0.062	0.004	0.083	0.934
Relationship orientation	0.063	0.109	1.926	0.049 *

Note: * *p* < 0.05, *** *p* < 0.001.

**Table 4:** Summary of the hypothesis testing (*N* = 336).

Hypothesis	Path	*β*	Sig.
H1	online health information experience→Health information sharing	0.417	*p* < 0.001
H2	Health information credibility→Health information sharing	−0.009	n.s
H3-1	relationship orientation→Health information sharing	0.109	*p* < 0.05
H3-2	authority orientation→Health information sharing	0.119	n.s
H3-3	family orientation→Health information sharing	0.004	*p* < 0.05

Note: n.s. stands for non-significance.
